# Non-Apnea Sleep Disorder associates with increased risk of incident heart failure—A nationwide population-based cohort study

**DOI:** 10.1371/journal.pone.0209673

**Published:** 2019-01-16

**Authors:** I-Duo Wang, Wu-Chien Chien, Chi-Hsiang Chung, Pei-Yi Tsai, Shan-Yueh Chang, Fan-Chun Meng, Chung-Kan Peng

**Affiliations:** 1 Division of Pulmonary and Critical Care Medicine, Department of Internal Medicine, Tri-Service General Hospital, National Defense Medical Center, Taipei, Taiwan; 2 School of Public Health, National Defense Medical Center, Taipei, Taiwan; 3 Department of Medical Research, Tri-Service General Hospital, Taipei, Taiwan; 4 Department of Radiation Oncology, National Cheng Kung University Hospital, Tainan, Taiwan; 5 Sleep Medicine Center, Tri-Service General Hospital, National Defense Medical Center, Taipei, Taiwan; National Yang-Ming University, TAIWAN

## Abstract

**Purpose:**

Non-apnea sleep disorder (NASD) increases the risk of cardiovascular events, such as hypertension and ischemic heart disease (IHD). Patients with heart failure (HF) are at higher risk for sleep disorder; however, there is no documentation on NASD’s association with HF to date. Therefore, our study aimed to determine whether NASD increases the risk of incident HF.

**Materials and methods:**

Using the outpatient and inpatient data from Taiwan’s Longitudinal Health Insurance Database, we conducted a nationwide cohort study of patients with a first-time diagnosis of NASD in the year 2000 and followed up the risk of incident heart failure until December 31, 2013. We calculated risks and incidence ratios of HF for patients with NASD compared with the general population. The cumulative incidence of NASD and the subsequent risk of HF are assessed by the Kaplan-Meier method and Cox regression using a matched comparison cohort of HF patients without NASD.

**Results:**

The NASD cohort had an adjusted hazard ratio (HR) of incident HF 19.7% higher than that of the cohort without NASD (95% CI = 1.130–1.270; p<0.001). In the NASD population, the mean interval to HF in males and females were 5.00±3.69 years and 5.00±3.66 years, respectively. The Kaplan-Meier analysis indicated that after the seventh year, the incidence of HF was higher in the NASD cohort than in the control cohort till the end of the follow up.

**Conclusions:**

Our study demonstrates that NASD patients are associated with a higher risk of incident HF.

## 1. Introduction

Sleep disorders (SDs) affect the quality of life of the general population in modern society [1]. Sleep disorders are a group of diseases characterized by the presence of disturbances in the amount, quality, or timing of sleep, or in the disturbance of physiological conditions associated with sleep [2]. According to the International Classification of Sleep Disorders (ICSD-3; American Academy of Sleep Medicine [AASM], 2014), SD has seven categories: insomnia, sleep-related breathing disorders, central disorders of hypersomnolence, circadian rhythm sleep disorders, parasomnias, sleep-related movement disorders, and other sleep disorders [2].

In the previous literature, obstructive sleep apnea (OSA) increases the risk of cardiovascular and cerebrovascular events [3, 4]. However, recent studies have shown that non-apnea sleep disorder (NASD) is also associated with an increased risk of hypertension, coronary artery disease (CAD), and ischemic stroke [5–7]. Heart failure (HF) is defined as a condition in which the heart loses the ability to pump enough blood to the body's tissues. Patients with HF are at a higher risk for SD, with an incidence rate between 23% and 73% [8, 9]. On the other hand, the correlation between SD and subsequent HF has rarely been documented. Laugsand et al. demonstrated an association between insomnia and the risk of incident heart failure in the Nord-Trøndelag Health study (HUNT) [10], but currently there is no documentation of the association between NASD and HF.

In the present study, we conducted the largest and the longest follow up period longitudinal nationwide population-based cohort study to date discussing the association between NASD and HF. We used Taiwan National Health Insurance Research Database (NHIRD) to investigate whether NASD increases the subsequent risk of HF while taking several cardiovascular risk factors, comorbidities, and demographic features into consideration.

## 2. Methods

### 2.1. Data source

Our results were obtained from the Taiwan National Health Insurance Research Database (NHIRD). Taiwan started the National Health Insurance (NHI) program in 1995. It covers approximately 99% of the 23.74 million individuals in the general population and has contracts with 97% of the hospitals and clinics in Taiwan [11]. The NHIRD includes information on medical facility registries, medication prescriptions, and outpatient, inpatient, and emergency visiting data for Taiwan’s general public. It offers representative data for medical and health-related research fields due to its high accuracy and validity [12, 13]. The NHIRD encrypts the identification numbers of all NHIRD records to ensure the privacy of all persons registered in the program. Additionally, our study protocol was also approved by the Institutional Review Board of the Tri-Service General Hospital (TSGHIRB No.2-105-05–082).

### 2.2. Participants

**Fig 1:**
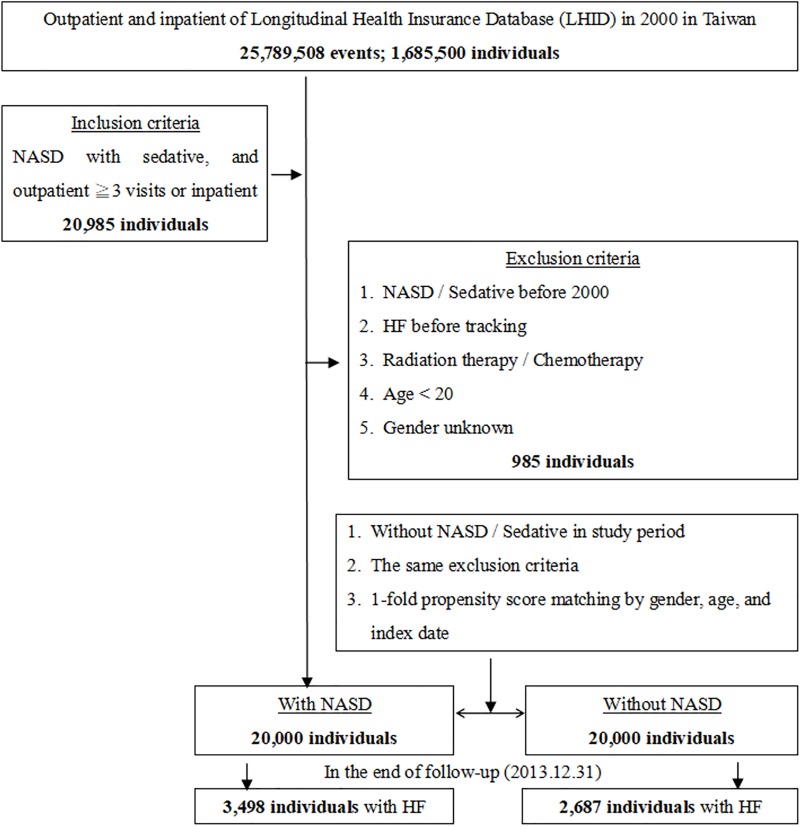
Flowchart of study sample selection from National Health Insurance Research Database in Taiwan.

This cohort study was based on the cumulative outpatients and inpatients population of 1,685,500 from Taiwan’s Longitudinal Health Insurance Database in the year 2000 (LHID; 2000) following from 2000 to 2013. The data were extracted according to the International Classification of Diseases, Ninth Revision, Clinical Modification (ICD-9-CM). Patients newly diagnosed with NASD under sedative usage who visited hospitals as outpatients more than 3 times, or who were hospitalized were included, for a total of 20,985 individuals. The date of diagnosis of NASD was used as the index date, and patients were followed until the appearance of heart failure (ICD-9-CM: 428). We defined the use of sedative (benzodiazepines and Z-drugs) by following the Anatomic Therapeutic Chemical classification system codes: N05BA and N05CD for benzodiazepines and N05CF for Z-drugs. The benzodiazepines enrolled in this study were diazepam, nitrazepam, chlordiazepoxide, clobazam, oxazepam, alprazolam, lorazepam and temazepam, whereas then Z-drugs included zopiclone and zolpidem. We excluded patients with sleep apnea, NASD diagnosis prior to the year 2000, diagnosis of heart failure prior to the index date, age less than 20 years old, patients who have received radiation therapy (ICD-9-CM V58.0) or chemotherapy (ICD-9-CM V58.1), and patients with unknown gender (Fig 1). NASD included sleep disturbances, unspecified (ICD-9-CM code: 780.50); other insomnia (ICD-9-CM code: 780.52); other hypersomnia (ICD-9-CM code: 780.54); disruptions of the 24-hour sleep-wake cycle (ICD-9-CM code: 780.55); dysfunctions associated with sleep stages or arousal from sleep (ICD-9-CM code: 780.56); unspecified sleep-related movement disorder (ICD-9-CM code 780.58); other sleep disturbances (ICD-9-CM code: 780.59), specific disorders of sleep of nonorganic origin (ICD-9-CM code: 307.4) and parasomnia (ICD-9-CM code: 327.44). Participants who matched our criteria were assigned to the study group. For each NASD patient, 1 comparison patient that was matched by gender, age, index date, and comorbidities was added to the control cohort. Common comorbidities, such as hypertension (ICD-9-CM code: 401–405), type 2 diabetes mellitus (DM; ICD-9-CM code: 250), hyperlipidemia (ICD-9-CM code: 272), chronic kidney disease (CKD; ICD-9-CM code: 585), ischemic heart disease (IHD; ICD-9-CM code: 410–414), stroke (ICD-9-CM code: 430–438), obesity (ICD-9-CM code: 278), anxiety (ICD-9-CM code: 300.00), and depression (ICD-9-CM code: 296.2–296.3, 300.4), were included to compare the baseline characteristics between the two groups.

### 2.3. Statistical analysis

The demographic features and common comorbidities between the study and comparison cohorts were compared using chi-squared tests. The mean ages of the cohorts were compared using a Student's t-test. We assessed the incidence (per 10^5^ person-years) of age, sex, and common comorbidities of HF for both the NASD and the control cohorts using Cox proportional hazard regression analysis to estimate the hazard ratios (HRs) with 95% confidence intervals (CIs). Each variant was adjusted for demographic characteristics and common comorbidities. The Kaplan-Meier method was used to evaluate the cumulative incidence of NASD and subsequent HF. All analyses were conducted using SPSS 22.0 software, with p< 0.05 considered statistically significant for the two-tailed tests.

## 3. Results

The study included 20,000 patients with NASD diagnosed in the year 2000 and 20,000 patients in the control cohort. The study was conducted between 2000 and 2013. The mean follow-up time was 10.36 ± 4.06 and 10.52 ± 3.78 years for the NASD and comparison cohorts, respectively (S1 Table). At the end of follow-up, 3,498 participants (17.49%) in the NASD cohort developed HF, compared to 2,687 participants in the control cohort (13.46%; p <0.001; Fig 1). Females were dominant in the study population (59.88%) and 41.66% of patients were younger than 50 years of age. The distribution of age and sex were similar between the study and control cohorts. The baseline data are listed in the Table 1 while the follow-up results of the demographic characteristics and common comorbidities are shown in Table 2.

**Table 1:** Characteristics of study in the baseline.

	With NASD (n = 20,000)		Without NASD (n = 20,000)		*P*
Characteristics	n	%	n	%	
Gender					0.999
Male	8,025	40.13	8,025	40.13	
Female	11,975	59.88	11,975	59.88	
Age (yrs)	53.27 ± 14.29		53.09 ± 16.08		0.237
Age group					0.999
20–49 yrs	8,332	41.66	8,332	41.66	
50–64 yrs	5,999	30.00	5,999	30.00	
≧65 yrs	5,669	28.35	5,669	28.35	
Comorbidities					
Hypertension	3,588	17.94	3,200	16.00	<0.001***
DM	1,174	5.87	716	3.58	<0.001***
Hyperlipidemia	400	2.00	308	1.54	<0.001***
CKD	46	0.23	41	0.21	0.668
IHD	808	4.04	372	1.86	<0.001***
Stroke	566	2.83	281	1.41	<0.001***
Obesity	7	0.04	4	0.02	0.549
Anxiety	1,522	7.83	555	2.81	<0.001***
Depression	1,598	8.22	611	3.10	<0.001***

*P*: Chi-square / Fisher exact test for the category variable and t-test for the continue variable.

*P < 0.05

**P < 0.01

***P < 0.001

Chronic kidney disease (CKD) is a condition characterized by a gradual loss of kidney function over time. Ischemic heart disease (IHD) refers to insufficient blood supply to the coronary arteries.

**Table 2:** Characteristics of study in the endpoint.

	With NASD (n = 20,000)		Without NASD (n = 20,000)		*P*
Characteristics	n	%	n	%	
HF	3,498	17.49	2,687	13.46	<0.001***
Gender					0.999
Male	8,025	40.13	8,025	40.13	
Female	11,975	59.88	11,975	59.88	
Age (yrs)	61.88 ± 17.14		62.27 ± 16.33		0.020*
Age group					<0.001***
20–49 yrs	4,624	23.12	5,411	27.06	
50–64 yrs	7,027	35.14	4,862	24.31	
≧65 yrs	8,349	41.75	9,727	48.64	
Comorbidities					
Hypertension	3,832	19.16	3,200	16.16	<0.001***
DM	1,89	9.47	1,587	7.94	<0.001***
Hyperlipidemia	984	4.92	930	4.65	0.214
CKD	543	2.72	402	2.01	<0.001***
IHD	1,361	6.81	1,082	5.41	<0.001***
Stroke	940	4.70	741	3.71	<0.001***
Obesity	10	0.05	9	0.05	0.819
Anxiety	1,677	8.39	617	3.09	<0.001***
Depression	1,791	8.96	790	3.95	<0.001***

*P*: Chi-square / Fisher exact test for the category variable and t-test for the continue variable.

*P < 0.05

**P < 0.01

***P < 0.001

**Fig 2:**
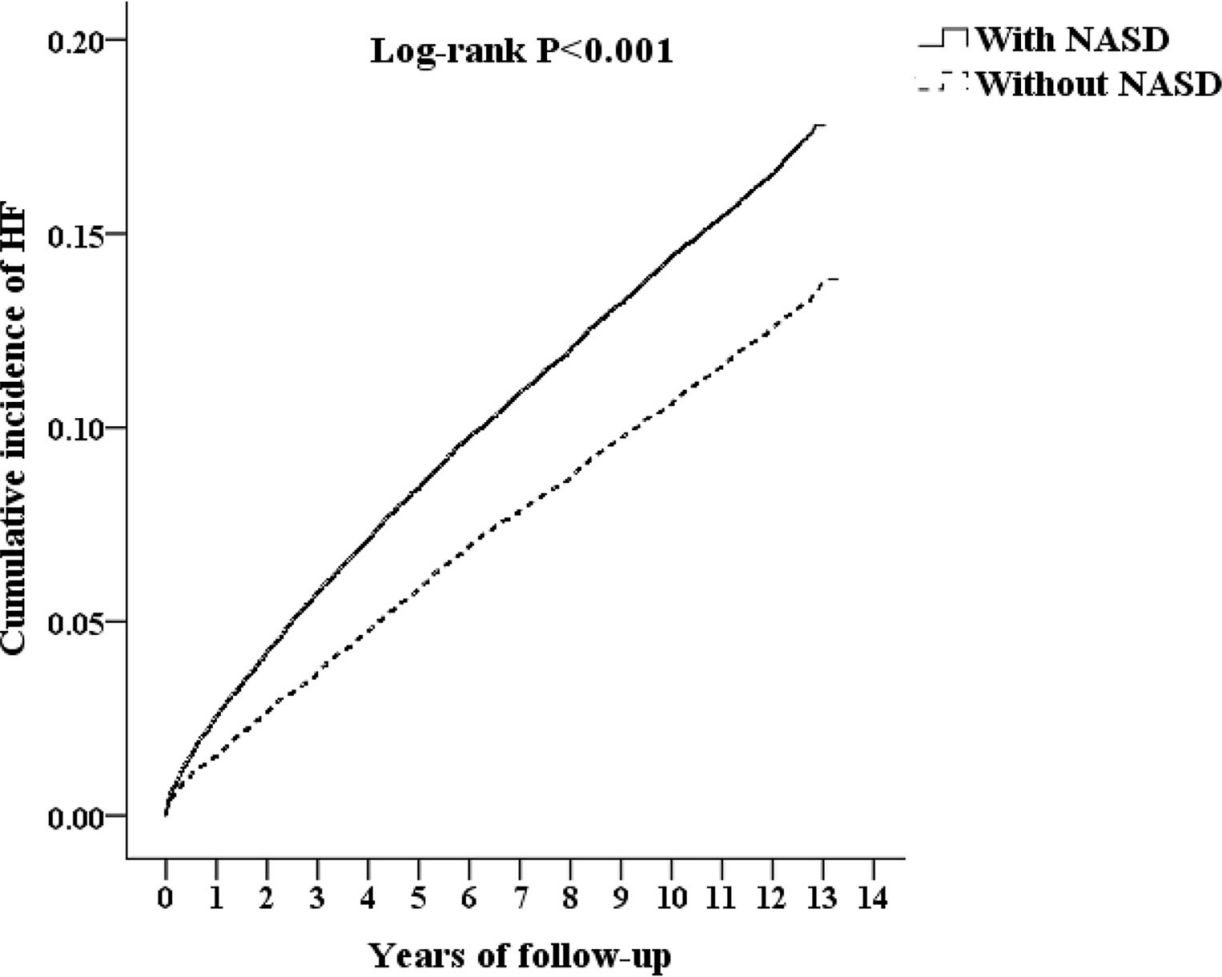
The Kaplan-Meier analysis for cumulative incidence of HF among patients aged over 20 years with and without NASD.

During the follow-up period, women had a higher percentage of HF in both cohorts. The average age was 61.88 ± 17.14 and 62.27 ± 16.33 years for the study and the comparison cohorts, respectively (p < 0.001; Table 2). The incidence of HF increased with age in both cohorts, with 41.75% of the patients aged 65 years had HF (p = 0.02; Table 2). Compared with the control cohort, the NASD cohort had a higher risk of HF and a higher prevalence of hypertension, DM, CKD, IHD, stroke, anxiety, and depression (p<0.001; Table 2). Kaplan-Meier analysis indicated that in the seventh year after diagnosis, the NASD cohort had a higher incidence of HF than the general population cohort (p = 0.036) and this difference persisted until the end of follow-up (log-rank p<0.001; Fig 2). The average time interval to HF was 5.00±3.62 and 5.62±3.68 years in the NASD population and the population without NASD, respectively. In the NASD population, the average time duration to HF was 5.00±3.69 years in males and 5.00±3.66 years in females (S2 Table).

Patients with NASD had a higher risk of HF compared with the control cohort (crude HR = 1.289 [95% CI, 1.226–1.356]; p<0.001). After adjusting for cardiovascular and other concomitant comorbidities, the HR of developing HF for patients with NASD decreased to 1.197 (95% CI = 1.130–1.270; p<0.001; Table 3). The influence of age on HF was significant, with an adjusted HR for HF of 3.639 (95% CI = 2.894–3.978; p<0.001) in the ≧65-year age group and an adjusted HR for HF of 2.466 (95% CI = 2.050–2.898; p<0.001) in the 50-to 64-year age group, compared to the 20-to 49-year age group. Additionally, the multivariable Cox regression model also showed that comorbidities, including hypertension, DM, IHD, anxiety, and depression are associated with an increased risk of HF (all p<0.05; Table 3). IHD had the highest HR for HF, with an adjusted HR = 3.601 (95% CI = 2.917–4.001; p<0.001), followed by hypertension (adjusted HR = 1.600 [95% CI, 1.301–1.874]; p<0.001). Psychological disorders such as anxiety and depression were associated with increased risk for HF, with adjusted HRs of 1.218 (95% CI = 1.016–2.514; p = 0.031) and 1.385 (95% CI = 1.107–1.662; p<0.001), respectively.

**Table 3:** Cox regression analysis for the risk of incident HF with interaction of comorbidities.

Characteristics	Crude HR	95% CI	*P*	Adjusted HR	95% CI	*P*
NASD *(reference*: *without)*	1.289	1.226–1.356	<0.001***	1.197	1.130–1.270	<0.001***
Male *(reference*: *female)*	1.004	0.954–1.056	0.189	0.964	0.852–1.032	0.068
Age group *(reference*: *20–49 yrs)*						
50–64 yrs	2.904	2.572–3.280	<0.001***	2.466	2.050–2.898	<0.001***
≧65 yrs	5.481	4.901–6.129	<0.001***	3.639	2.894–3.978	<0.001***
Comorbidities *(reference*: *without)*						
Hypertension	2.065	1.951–2.858	<0.001***	1.600	1.301–1.874	<0.001***
DM	1.214	1.111–1.312	<0.001***	1.019	1.001–1.521	0.049*
Hyperlipidemia	0.478	0.365–0.592	<0.001***	0.383	0.201–0.655	<0.001***
CKD	1.401	1.183–1.609	<0.001***	1.151	0.972–1.436	0.074
IHD	5.281	4.175–6.778	<0.001***	3.601	2.917–4.001	<0.001***
Stroke	0.702	0.600–0.845	0.001**	0.598	0.312–0.772	<0.001***
Obesity	1.123	0.356–2.987	0.643	1.244	0.527–3.411	0.726
Anxiety	1.323	1.025–2.633	0.027*	1.218	1.016–2.514	0.031*
Depression	1.429	1.125–1.774	<0.001***	1.385	1.107–1.662	<0.001***

HR = hazard ratio, CI = confidence interval, Adjusted HR: Adjusted variables listed in the table

*P < 0.05

**P < 0.01

***P < 0.001

The overall incidence rate of HF in NASD was 1,404.45 per 10^5^ person-years (Table 4). Men and women were both affected by NASD with the incidence rate of 1,441.92 in males and 1,380.18 in females per 10^5^ person-years. When adjusted for other covariates, females had a higher HR (adjusted HR = 1.198 [95% CI, 1.134–1.286]; p<0.001) for HF compared to males (adjusted HR = 1.195 [95% CI, 1.128–1.268]; p = 0.001; Table 4). The incidence of HF increased with age in both cohorts. Also, NASD had a higher impact of HF in the age group ≧65 years with the adjusted HR 1.269 (95% CI = 1.140–1.345; p<0.001) compared to patients without NASD (Table 4). The risk of HF in NASD patients increased with concomitant comorbidities. Hypertension, DM, hyperlipidemia, CKD, IHD, stroke and obesity were associated with higher risk of HF in NASD patients compared to the patients without NASD (all p<0.05; Table 4).

**Table 4:** Incidence and HRs of incident HF for NASD cohort compared with non-NASD cohort by demographic characteristics and comorbidities.

		With NASD (n = 20,000)			Without NASD (n = 20.000)			With vs. Without NASD *(reference)*		
Stratified		HF events	PYs	Rate	HF events	PYs	Rate	Adjusted HR	95% CI	*P*
Overall		3,498	249,065.12	1,404.45	2,687	254,381.96	1,056.29	1.197	1.130–1.270	<0.001***
Gender	Male	1,412	97,925.13	1,441.92	1,093	100,731.24	1,085.07	1.195	1.128–1.268	0.001**
	Female	2,086	151,139.99	1,380.18	1,594	153,650.72	1,037.42	1.198	1.134–1.286	<0.001***
Age group	20–49 yrs	145	60,869.76	238.21	141	56,035.70	251.63	1.065	1.005–1.165	0.045*
	50–64 yrs	472	69,242.99	681.66	492	84,890.32	579.57	1.184	0.894–1.208	0.126
	≧65 yrs	2,881	118,952.37	2,421.98	2,054	113,455.93	1,810.39	1.269	1.140–1.345	<0.001***
Hypertension	With	2,626	214,830.01	1,222.36	2,032	219,930.98	923.93	1.186	1.099–1.254	0.003**
	Without	872	34,235.11	2,547.09	655	34,450.98	1,901.25	1.245	1.137–1.801	<0.001***
DM	With	3,187	228,913.26	1,392.23	2,465	233,404.54	1,056.11	1.122	1.007–1.265	0.040*
	Without	311	20,151.86	1,543.28	222	20,977.42	1,058.28	1.345	0.865–2.978	0.266
Hyperlipidemia	With	3,428	234,467.14	1,462.04	2,622	237,402.76	1,104.45	1.194	1.098–1.801	0.003*
	Without	70	14,597.98	479.52	65	16,979.20	382.82	1.220	0.826–1.975	0.595
CKD	With	3,397	243,293.53	1,396.26	2,618	249,068.99	1,051.11	1.192	1.101–1.365	0.001**
	Without	101	5,771.59	1,749.95	69	5,312.97	1,298.71	1.273	0.744–2.014	0.348
IHD	With	2,518	231,601.00	1,087.21	2,256	244,286.74	923.50	1.060	1.025–1.245	0.027*
	Without	980	17,464.12	5,611.51	431	10,095.22	4,269.35	1.183	1.000–1.336	0.050*
Stroke	With	3,408	239,400.89	1,423.55	2,632	246,299.71	1,068.62	1.199	1.101–1.257	0.007*
	Without	90	9,664.23	931.27	55	8,082.25	680.50	1.234	0.567–2.438	0.486
Obesity	With	3,498	248,890.83	1,405.44	2,687	254,281.41	1,056.70	1.197	1.130–1.270	<0.001***
	Without	0	174.29	0	0	100.55	0	-	-	-
Anxiety	With	3,298	239,089.50	1,379.40	2,589	245,638.45	1,053.99	1.178	0.672–1.540	0.412
	Without	200	9,975.62	2,004.89	98	8,743.51	1,120.83	1.645	1.428–2.001	<0.001***
Depression	With	3,243	239,066.40	1,356.53	2,580	245,136.56	1,052.47	1.160	0.611–1.513	0.387
	Without	255	9,998.72	2,550.33	107	9,245.40	1,157.33	1.978	1.702–2.256	<0.001***

PYs = person-years, Rate = incidence (per 10^5^ PYs), Adjusted HR = adjusted hazard ratio: adjusted variables listed in Table 3, CI = confidence interval

*P < 0.05

**P < 0.01

***P < 0.001

The risk of different types of NASD on incident HF is presented in Table 5. Both the insomnia and the non-insomnia groups were associated with a risk of heart failure. Among patients with NASD, insomnia patients accounted for an adjusted HR with a 36.5% increased risk of heart failure (95% CI = 1.185–1.784; p<0.001) whereas in non-insomnia patients, the adjusted risk of heart failure was 1.199 (95% CI = 1.100–1.420; p = 0.001). In the non-insomnia group, patients with unspecified sleep-related movement disorder and other sleep disturbances were associated with adjusted risks of 2.764 (95% CI = 1.682–3.895; p<0.001) and 2.053 (95% CI = 1.298–3.112; p<0.001), respectively.

**Table 5:** Incidence rates and HRs of incident HF in patients with different types of NASDs.

Types of NASDs	HF events	PYs	Rate	Adjusted HR	95% CI	*P*
Without NASD (n = 20,000)	2,687	254,381.96	1,056.29	*Reference*		
With NASD (n = 20,000)	3,498	249,065.12	1,404.45	1.197	1.130–1.270	<0.001***
Insomnia (n = 13,851)	2,211	140,170.65	1,577.36	1.365	1.185–1.784	<0.001***
Non-insomnia (n = 6,149)	1,287	108,894.47	1,181.88	1.199	1.100–1.420	0.001**
Sleep disturbances (n = 2,875)	416	40,977.23	1,015.20	0.988	0.477–1.945	0.489
Hypersomnia (n = 81)	1	9,195.12	10.88	0.008	0.001–18.842	0.845
Disruptions of 24-hour sleep-wake cycle (n = 6)	0	31.27	0	0	-	0.901
Dysfunctions associated with sleep stages or arousal from sleep (n = 51)	3	5,394.10	55.62	0.041	0.005–11.775	0.810
Unspecified sleep-related movement disorder (n = 33)	225	7,258.93	3,099.63	2.764	1.682–3.895	<0.001***
Other sleep disturbances (n = 675)	308	11,256.74	2,736.14	2.053	1.298–3.112	<0.001***
Specific non-organic sleep disorder (n = 2,100)	313	31,656.11	988.75	0.902	0.415–1.901	0.342
Parasomnia (n = 31)	0	1.87	0	0	-	0.945
Sleep-related movement disorders (n = 297)	21	3,123.10	672.41	0.645	0.347–1.864	0.475

PYs = person-years, Rate = incidence (per 10^5^ PYs), Adjusted HR = adjusted hazard ratio: adjusted variables listed in Table 3, CI = confidence interval.

*P < 0.05

**P < 0.01

***P < 0.001

## 4. Discussion

Our study is the largest nationwide cohort study to date discussing the association between NASD and incident heart failure. The mean follow-up time in the NASD cohort was 10.36 ± 4.06 years. Previous articles in the literature have only focused on the correlation between insomnia and the risk of heart failure. Laugsand et al., who had a mean follow-up of 11.3 years, showed that the number of insomnia symptoms cumulatively increased the risk of incident HF [10]. Two other small studies also investigated the association of insomnia and incident heart failure. Ingelsson et al. [14] studied 2,314 middle-aged men prospectively over 30 years of follow-up. Newman [15] discovered that daytime sleepiness predicted incident HF and cardiovascular mortality in a 5-year follow-up. However, none of them described an association between NASD and incident HF. In our study, the overall incidence ratio of incident HF was 33% higher in the NASD cohort than in the control cohort. The crude HR in the study cohort was 1.289. After adjusting for cardiovascular and other concomitant comorbidities, the HR decreased to 1.197, indicating that gender, age, and other covariates had an interaction with NASD, leading to an increased risk of HF.

Women were predominant in our NASD study, making up 59.88% of the population. This has also been shown in other studies, suggesting that females are more likely to have NASD than males [16, 17]. After adjusting for age and other comorbidities, women with NASD had a higher risk of developing HF than men (adjusted HR of 1.198 in women and 1.195 in men). Our result was similar to the findings of Laugsand LE et al in the Nord-Trøndelag Health study (HUNT) [10]. However, they did not calculate the time duration between NASD diagnosis and the development of HF. In this study, the average time interval to HF was 5.00 ± 3.62 years in the NASD population, which was similar to the finding from the Kaplan-Meier analysis showing that after seven years, NASD patients had a higher risk of developing HF than the control cohort. There was no significant difference in the average time duration to HF in males and females (5.00 ± 3.69 and 5.00 ± 3.66 years, respectively).

Age is another important risk factor for HF. Patients aged ≧65 years carried 5.481 times the risk for HF, while participants in the 50- to 64-year age group had a 2.904-fold risk, compared to the 20- to 49-year-olds. However, when adjusting for gender and other concomitant comorbidities, the HR decreased to 3.639 and 2.466 times for the ≧65-year age group and the 50- to 64-year age group, respectively. This finding can be explained by the presence of more comorbidities in older adults that may interact with NASD, leading to the development of HF.

The pathophysiology of NASD and its association with HF has not yet been fully understood. Insomnia and other NASDs are disorders of hyperarousal that are accompanied by activation of stress hormones and increased activity of the hypothalamic-pituitary-adrenal (HPA) axis and sympathetic nervous system [18]. The activated stress response increases heart rate, blood pressure, and the secretion of pro-inflammatory cytokines and catecholamines, but it decreases heart rate variability [19]. Patients with NASD are associated with an unhealthy lifestyle and concomitant comorbidities, including hypertension, type 2 diabetes, CAD, CKD, and ischemic stroke [5–7, 20, 21]. This may lead to endothelial dysfunction, atherosclerosis, and progressive left ventricular remodeling [10], leading to subsequent HF. In our study, we found that both the insomnia and non-insomnia groups were associated with a risk of heart failure. The insomnia patients had a 36.5% increased risk of heart failure, compared to a 19.9% increased risk in the non-insomnia group patients.

Psychological distress, such as anxiety and depression, is an important factor for HF that may have confounding interactions with NASD, thereby increasing the risk of HF. However, in previous studies, the association between insomnia and cardiovascular disease was not adjusted for these psychological factors [22]. In our study, we have adjusted for many covariates, including gender, age, and cardiovascular risk factors, and studied the impact of anxiety and depression on HF. Anxiety alone increased the risk of HF by 21.8%, while depression was associated with a 38.5% increase.

Our study have several limitations. First, we could not obtain detailed patient information such as smoking habits, alcohol consumption, physical activity, socioeconomic status, or family history from the NHIRD. Second, there were selection bias while using the NHIRD; thus resulting in the low numbers of some diagnosis in the NASD categories. Third, there were no objective measurements of NASD in this study, nor did we use polysomnography. Despite the limitations, this study provides a nationwide population-based cohort on the association between NASDs and the risk of incident heart failure.

In conclusion, we have identified that NASD increases the risk of incident heart failure. An increasing number of people suffer from NASD in modern society; therefore, we believe that our study can act as a wake-up call for clinical practitioners as well as for the general public in the prevention of cardiovascular events.

## Supporting information

S1 Table Years of follow-up. (DOC)

S2 Table Years to HF. (DOC)
