# Supplementary material for: Non-Apnea Sleep Disorder associates with increased risk of incident heart failure—A nationwide population-based cohort study
Source: PLoS One. 2019 Jan 16;14(1):e0209673. doi: 10.1371/journal.pone.0209673 (PMC6334965; doi:10.1371/journal.pone.0209673)
Supplement: S1 Table — (DOC) [file pone.0209673.s001.doc]

**S1 Table.** Years of follow-up

|  | **Min** | **Medium** | **Max** | **Mean** |
| --- | --- | --- | --- | --- |
| Overall | 0.01 | 12.47 | 13.75 | 10.44 ± 3.84 |
| With NASD (n = 20,000) | 0.01 | 12.13 | 13.10 | 10.36 ± 4.06 |
| Without NASD (n = 20,000) | 0.01 | 12.52 | 13.75 | 10.52 ± 3.78 |
