# Supplementary material for: Non-Apnea Sleep Disorder associates with increased risk of incident heart failure—A nationwide population-based cohort study
Source: PLoS One. 2019 Jan 16;14(1):e0209673. doi: 10.1371/journal.pone.0209673 (PMC6334965; doi:10.1371/journal.pone.0209673)
Supplement: S2 Table — (DOC) [file pone.0209673.s002.doc]

**S2 Table.** Average time duration to HF in NASD patients

|  | **With NASD** (n = 20,000) | | | |  | **Without NASD** (n = 20,000) | | | |
| --- | --- | --- | --- | --- | --- | --- | --- | --- | --- |
| Gender | **Min** | **Medium** | **Max** | **Mean** |  | **Min** | **Medium** | **Max** | **Mean** |
| Overall | 0.01 | 4.48 | 12.83 | 5.00 ± 3.62 |  | 0.01 | 5.41 | 12.93 | 5.62 ± 3.68 |
| Male | 0.01 | 4.31 | 12.79 | 5.00 ± 3.69 |  | 0.01 | 5.20 | 12.95 | 5.59 ± 3.78 |
| Female | 0.01 | 4.39 | 12.83 | 5.00 ± 3.66 |  | 0.01 | 5.31 | 12.95 | 5.60 ± 3.74 |
